# Semaglutide and cardiovascular outcomes by baseline HbA_1c_ in diabetes: the SUSTAIN 6 and PIONEER 6 trials

**DOI:** 10.1093/eurheartj/ehae028

**Published:** 2024-02-28

**Authors:** Linda G Mellbin, Deepak L Bhatt, Jens-Peter David, Kathrine Ekström, Mark C Petrie, Søren Rasmussen, Tina Vilsbøll

**Affiliations:** Department of Medicine Solna, Karolinska Institutet, Eugeniavägen 27, 171 64 Solna, Stockholm, Sweden; Heart Vascular and Neuro Theme, Karolinska University Hospital, K2 Medicin, K2 Kardio Pernow J, 171 77 Solna, Stockholm, Sweden; Mount Sinai Fuster Heart Hospital, Icahn School of Medicine at Mount Sinai, New York, NY, USA; Novo Nordisk A/S, Søborg, Denmark; Novo Nordisk A/S, Søborg, Denmark; School of Cardiovascular and Metabolic Health, University of Glasgow, Glasgow, UK; Novo Nordisk A/S, Søborg, Denmark; Clinical Research, Steno Diabetes Center Copenhagen, University of Copenhagen, Copenhagen, Denmark

**Keywords:** Cardiovascular outcomes trial, Glucagon-like peptide-1 receptor agonist, HbA_1c_, Major adverse cardiovascular events, PIONEER 6, *Post hoc* analysis, Semaglutide, SUSTAIN 6, Type 2 diabetes

## Introduction

Certain glucagon-like peptide-1 receptor agonists reduce major adverse cardiovascular events (MACE) compared with placebo in people with type 2 diabetes at high cardiovascular (CV) risk,^1^ and diabetes and cardiology guidelines recommend their use (or that of sodium–glucose co-transporter 2 inhibitors) in this population regardless of baseline glycated haemoglobin (HbA_1c_).^2,3^

This *post hoc* analysis of the SUSTAIN 6 (once-weekly subcutaneous [s.c.] semaglutide)^4^ and PIONEER 6 (once-daily oral semaglutide)^5^ CV outcomes trials aimed to evaluate the treatment effect of the glucagon-like peptide-1 analogue semaglutide vs. placebo on MACE by baseline HbA_1c_.

## Methods

Data were pooled for participants with type 2 diabetes and established CV disease or high CV risk in the SUSTAIN 6 and PIONEER 6 trials. Participants received s.c. semaglutide (0.5 or 1.0 mg)/oral semaglutide (14 mg) or volume-matched placebo; detailed trial descriptions can be found elsewhere.^4,5^

The primary outcome for both trials was time to first MACE: a composite of CV death, non-fatal myocardial infarction (MI), or non-fatal stroke. Secondary outcomes included time to occurrence of individual MACE components.

Additional efficacy outcomes included change from baseline in HbA_1c_ and body weight to Week 80 for SUSTAIN 6 and Week 83 in PIONEER 6 (final study visit), which were the visits closest in each trial.

### Statistical analysis

Continuous variables are presented as means unless otherwise indicated. A quadratic spline function of baseline HbA_1c_ by treatment was used to analyse treatment effect on time to first MACE across a continuum of baseline HbA_1c_ values in a Cox proportional hazards model. Linear splines were used to analyse treatment effect across individual MACE components due to the low number of events.

Time to first MACE and its components were also compared between baseline HbA_1c_ subgroups [<8% and ≥8% (<64 and ≥64 mmol/mol)]; cut-offs were selected close to the median in a Cox proportional hazards model, with treatment by subgroup as a fixed factor. Key predictors of CV-renal disease at baseline were added as covariates: sex, glucose-lowering therapy, smoking, previous stroke or MI, region, age, diabetes duration, estimated glomerular filtration rate, and continuous HbA_1c_. The subgroup analysis comparing HbA_1c_ of <8% and ≥8% (<64 and ≥64 mmol/mol) was adjusted based on these predictors using inverse probability weighting.

Heterogeneity in treatment effect across HbA_1c_ of <8% and ≥8% (<64 and ≥64 mmol/mol) subgroups was indicated by interaction *P*-values, with *P* < .05 indicating a significant interaction. No adjustment for multiplicity was performed.

Estimated treatment differences (ETDs) in change in HbA_1c_ and body weight from baseline with semaglutide vs. placebo across baseline HbA_1c_ values were assessed using a mixed model with the quadratic spline of baseline HbA_1c_.

### Ethics

This study was conducted in accordance with the Declaration of Helsinki and the Guidelines for Good Pharmacoepidemiology Practices, and approved by the institutional review boards and ethics committees for each participating centre. All participants provided written informed consent to participate in the SUSTAIN 6 (NCT01720446) and PIONEER 6 (NCT02692716) trials.

## Results

### Baseline characteristics

Of the 6480 participants included in the analysis, 3239 received semaglutide and 3241 received placebo (followed for a median of 2.1 years in SUSTAIN 6 and 15.9 months in PIONEER 6). At baseline, mean age was 65.4 years, 64.5% were male, 44.5% had experienced a previous CV event, mean diabetes duration was 14.4 years, and mean HbA_1c_ was 8.4% (69 mmol/mol). Detailed baseline characteristics for each individual trial are available elsewhere.^4,5^

### Impact on cardiovascular outcomes

**Figure 1:**
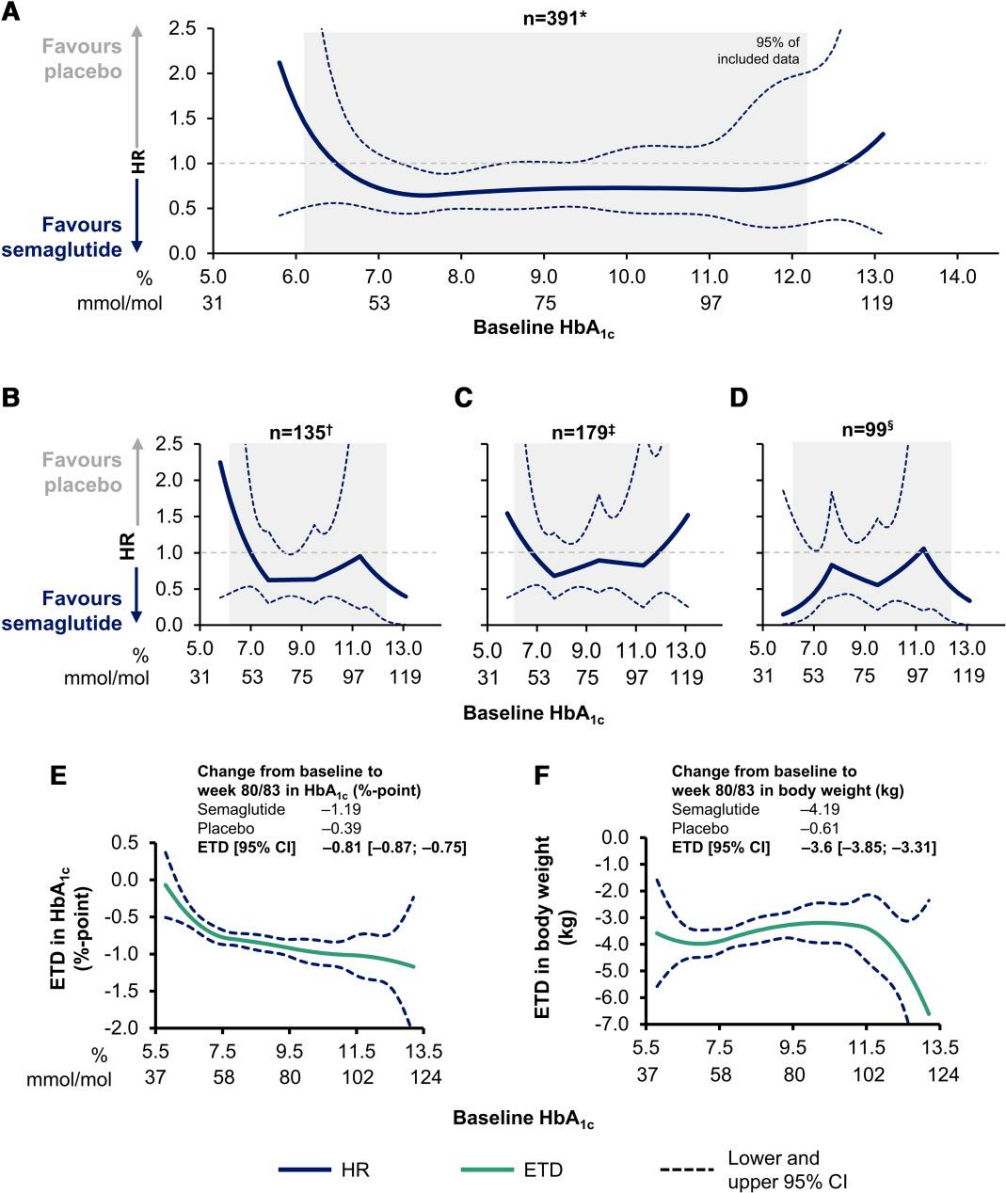
Risk of (*A*) major adverse cardiovascular events, (*B*) cardiovascular death, (*C*) non-fatal myocardial infarction, and (*D*) non-fatal stroke by baseline HbA_1c_, and estimated treatment difference in HbA_1c_ (*E*) and body weight (*F*) with semaglutide vs. placebo across baseline HbA_1c_ values in the pooled SUSTAIN 6 and PIONEER 6 population. *n = 169 semaglutide; *n* = 222 placebo. ^†^*n* = 59 semaglutide; *n* = 76 placebo. One participant receiving semaglutide was not included owing to a missing HbA_1c_ value at baseline. ^‡^*n* = 84 semaglutide; *n* = 95 placebo. ^§^*n* = 39 semaglutide; *n* = 60 placebo. For the components of major adverse cardiovascular events, multiple events in the same participant were reported separately unlike overall major adverse cardiovascular events, which was time to first event. The lower and upper *x*-axis boundaries of the grey box correspond to the 2.5 and 97.5 percentiles (HbA_1c_ of >6.1% and <12.2% [>43 and <110 mmol/mol], respectively); therefore, 95% of the data are included in the grey box. Major adverse cardiovascular events were a composite of cardiovascular death, non-fatal myocardial infarction, and non-fatal stroke. Time to first occurrence of major adverse cardiovascular events was analysed using a Cox proportional hazards model with a quadratic spline function of baseline HbA_1c_ by treatment. Time to first occurrence of major adverse cardiovascular event components was analysed using a Cox proportional hazards model with a linear spline function of baseline HbA_1c_ by treatment. Change in HbA_1c_ and body weight at Week 80/83 was analysed using a mixed model with the quadratic spline of baseline HbA_1c_. CI, confidence interval; ETD, estimated treatment difference; HR, hazard ratio; *n*, number of participants with an event.

Major adverse cardiovascular events were experienced by 391 (6.0%) participants during the in-trial period; 169 (5.2%) events occurred in the semaglutide and 222 (6.8%) in the placebo group. As previously published, the overall hazard ratio (HR) [95% confidence interval (CI)] was 0.76 [0.62; 0.92], with the largest effect seen for non-fatal stroke (HR 0.65 [0.43; 0.97]).^6^ Across a continuum of baseline HbA_1c_ values (6.5–12.6% [48–114 mmol/mol]), the HR for MACE favoured semaglutide compared with placebo (*Figure 1A*), with a similar trend observed for individual MACE components: 59 (1.8%) CV deaths, 84 (2.6%) non-fatal MIs, and 39 (1.2%) non-fatal strokes with semaglutide vs. 76 (2.3%), 95 (2.9%), and 60 (1.9%) with placebo, respectively (*Figure 1B–D*).

When comparing participants with HbA_1c_ <8% (<64 mmol/mol; *n* = 2826) vs. the ≥8% (≥64 mmol/mol) subgroup (*n* = 3626), CV deaths, non-fatal MIs, and non-fatal strokes occurred in 47 (1.7%) vs. 87 (2.4%), 66 (2.3%) vs. 113 (3.1%), and 37 (1.3%) vs. 62 (1.7%) participants, respectively.

In the adjusted analysis for MACE, for the baseline HbA_1c_ <8% (<64 mmol/mol) subgroup, HRs [95% CI] for MACE, CV death, non-fatal MI, and non-fatal stroke were 0.80 [0.57; 1.11], 0.87 [0.49; 1.56], 0.98 [0.60; 1.59], and 0.52 [0.26; 1.05], respectively, compared with 0.72 [0.56; 0.93] (*P*_interaction_ = .65), 0.70 [0.46; 1.07] (*P*_interaction_ = .55), 0.83 [0.57; 1.20] (*P*_interaction_ = .60), and 0.74 [0.44; 1.22] (*P*_interaction_ = .44) in the baseline HbA_1c_ ≥8% (≥64 mmol/mol) subgroup, indicating no significant difference in treatment effect between subgroups.

### Impact on metabolic outcomes

Semaglutide reduced HbA_1c_ and body weight from baseline to Week 80/83 vs. placebo, regardless of baseline HbA_1c_ (*Figure 1E* and *F*). When comparing HbA_1c_ subgroups, changes in HbA_1c_ in participants with baseline HbA_1c_ <8% (<64 mmol/mol; ETD −0.64 [95% CI −0.73; −0.55]) were less pronounced than those with HbA_1c_ ≥8% (≥64 mmol/mol; ETD −0.94 [95% CI −1.02; −0.86]; *P*_interaction_ < .001). Reductions in body weight were similar across HbA_1c_ subgroups (ETD −3.78 kg [95% CI −4.19; −3.38] for HbA_1c_ <8% [<64 mmol/mol], and ETD −3.45 kg [95% CI −3.81; −3.09] for HbA_1c_ ≥8% [≥64 mmol/mol]; *P*_interaction_ = .22).

## Conclusion

The present analyses support semaglutide use regardless of HbA_1c_ values, in line with current diabetes and cardiology guidelines.^2,3^ The analyses suggest that baseline HbA_1c_ values do not modify the treatment benefit of semaglutide vs. placebo on MACE; semaglutide reduced MACE across a continuum of baseline HbA_1c_, and a trend for risk reduction was observed for individual MACE components regardless of baseline HbA_1c_. In addition, semaglutide effects on blood glucose control and body weight over time are significantly different vs. placebo regardless of baseline HbA_1c_ levels.

## Declarations

### Data Availability

The data that support the findings of this study are available from the corresponding author upon reasonable request.

### Pre-registered Clinical Trial Number

SUSTAIN 6 (NCT01720446) and PIONEER 6 (NCT02692716) are registered with ClinicalTrials.gov.

## References

[1] Sattar N , Lee MMY, Kristensen SL, Branch KRH, Del Prato S, Khurmi NS, et al Cardiovascular, mortality, and kidney outcomes with GLP-1 receptor agonists in patients with type 2 diabetes: a systematic review and meta-analysis of randomised trials. Lancet Diabetes Endocrinol 2021;9:653–62. 10.1016/S2213-8587(21)00203-5 34425083

[2] Davies MJ , Aroda VR, Collins BS, Gabbay RA, Green J, Maruthur NM, et al Management of hyperglycaemia in type 2 diabetes, 2022. A consensus report by the American Diabetes Association (ADA) and the European Association for the Study of Diabetes (EASD). Diabetologia 2022;65:1925–66. 10.1007/s00125-022-05787-2 36151309 PMC9510507

[3] Marx N , Federici M, Schutt K, Müller-Wieland D, Ajjan RA, Antunes MJ, et al 2023 ESC guidelines for the management of cardiovascular disease in patients with diabetes. Eur Heart J 2023;44:4043–140. 10.1093/eurheartj/ehad192 37622663

[4] Marso SP , Bain SC, Consoli A, Eliaschewitz FG, Jódar E, Leiter LA, et al Semaglutide and cardiovascular outcomes in patients with type 2 diabetes. N Engl J Med 2016;375:1834–44. 10.1056/NEJMoa16071415 27633186

[5] Husain M , Birkenfeld AL, Donsmark M, Dungan K, Eliaschewitz FG, Franco SR, et al Oral semaglutide and cardiovascular outcomes in patients with type 2 diabetes. N Engl J Med 2019;381:841–51. 10.1056/NEJMoa1901118 31185157

[6] Husain M , Bain SC, Jeppesen OK, Lingvay I, Sørrig R, Treppendahl MB, et al Semaglutide (SUSTAIN and PIONEER) reduces cardiovascular events in type 2 diabetes across varying cardiovascular risk. Diabetes Obes Metab 2020;22:442–51. 10.1111/dom.13955 31903692 PMC7064975

